# Novel approaches for the serodiagnosis of louse-borne relapsing fever

**DOI:** 10.3389/fcimb.2022.983770

**Published:** 2022-09-20

**Authors:** Florian Röttgerding, John Njeru, Elif Schlüfter, Andreas Latz, Rouzbeh Mahdavi, Ulrich Steinhoff, Sally J. Cutler, Silke Besier, Volkhard A. J. Kempf, Volker Fingerle, Peter Kraiczy

**Affiliations:** ^1^ Institute of Medical Microbiology and Infection Control, University Hospital of Frankfurt, Goethe-University Frankfurt, Frankfurt, Germany; ^2^ Centre for Microbiology Research, Kenya Medical Research Institute, Nairobi, Kenya; ^3^ NovaTec Immundiagnostica GmbH, Dietzenbach, Germany; ^4^ Institute for Medical Microbiology and Hygiene, Philipps-University Marburg, Marburg, Germany; ^5^ School of Health, Sports & Bioscience, University of East London, London, United Kingdom; ^6^ National Reference Center for Borrelia, Bavarian Health and Food Safety Authority, Oberschleissheim, Germany

**Keywords:** spirochetes, borrelia, borrelia recurrentis, relapsing fever, louse-borne relapsing fever, serological diagnosis

## Abstract

Louse-borne relapsing fever (LBRF) caused by *B. recurrentis* is a poverty-related and neglected infectious disease with an endemic focus in the Horn of Africa. Re-emergence of the disease occurred in Europe during the refugee crisis in 2015 and sporadic outbreaks were frequently reported in Eastern Africa where poor settings lack affordable diagnostics. Currently, there are no validated *in vitro* assays available for the serodiagnosis of LBRF. The aim of this study was to develop novel and reliable immunoassays by investigating clinically suspected and culture-confirmed serum samples from LBRF patients and a broad panel of serum samples from patients with other spirochetal, bacterial, and parasitic diseases. We identified two immunoreactive antigens (complement-inhibiting protein CihC and the glycerophosphodiester phosphodiesterase GlpQ of *B. recurrentis*) as the most promising target candidates leading to the evaluation of two immunoassays (line immunoblot and ELISA) for IgM and IgG. To optimize the IgM immunoassay, we conducted a bioinformatic approach to localize the relevant immunogenic regions within CihC. By utilizing a N-terminal CihC fragment, the sensitivity and specificity of both immunoassays (CihC and GlpQ) were high (IgM: sensitivity 100%, specificity of 89.9%, IgG: sensitivity 100%, specificity 99.2%). In conclusion, our findings indicate the diagnostic potential of CihC and GlpQ as valuable markers for the serodiagnosis of LBRF even at early time points of infection. Here, we provide strong evidence for the utilization of these immunoassays as reliable tools in clinical practice.

## Introduction

Louse-borne relapsing fever (LBRF), a neglected vector-borne disease caused by *Borrelia (B.) recurrentis* (Craigie, 1843; Obermeier, 1873; Henderson, 2015; Warrell, 2019), was responsible for the major plagues in the 17th and 18th century whereby the latest epidemic outbreak occurred during two world wars in 1903-1936 and 1943-1946 (Mac Arthur, 1957; Bryceson et al., 1970; Cutler et al., 2009). Historically, LBRF was described as early as the 5th century BC by Hippocrates and was considered to cause the ´Yellow plague´ in 550 AD in Europe (Chadwick and William, 1950; Lloyd, 1983). Despite its historical significance and global distribution, LBRF disappeared in most parts of the world due to improved hygienic standards which are directly correlated with the disappearance of the only known vector, the human body louse *Pediculus humanus*. Certainly, the disease is associated with poverty, famine, war, political turmoil as well as natural disasters resulting in major refugee migrations (Cutler, 2010; Chikeka and Dumler, 2015). Although LBRF is currently geographically restricted to a few countries in the Horn of Africa including Ethiopia, Eritrea, Somalia, and South Sudan, sporadic outbreaks are frequently reported to adjacent territories (Cutler et al., 2009; Cutler, 2010; Barbour, 2011). Besides being an epidemic disease in restricted parts of Eastern Africa, recent re-emergence in Europe occurred during the refugee crisis in 2015 and 2016 with almost 100 clinically diagnosed LBRF cases (Warrell, 2019; Kahlig et al., 2021). These sporadic outbreaks, probably facilitated through overcrowded refugee camps, clearly illustrate the high potential of a rapid global spread of LBRF due to an increase in poor hygienic standards and disastrous living conditions.

If patients are left untreated, the case fatality rate of LBRF is high and exceed up to 30% (Bryceson et al., 1970; Meri et al., 2006; Warrell, 2019). Infection occurs *via* the transmission of the spirochete *B. recurrentis* by direct contact with either infectious hemocoel of crushed lice or feces through micro-lesions in the human skin, or even through intact mucous membranes (Bryceson et al., 1970). After 4-18 days, the patients develop a sudden fever up to 40°C which is accompanied by rigors, headache, dizziness, generalized aches, and pain (Bryceson et al., 1970; Larsson et al., 2009; Warrell, 2019). Antibiotic treatment can reduce the mortality rate to as low as 2-5% but an elevated risk for a severe Jarisch-Herxheimer reaction, which in itself can be lethal, occurs in 80% to 90% among these patients (Barbour, 2011). Due to the lack of epidemiological data, the impact of LBRF on high-risk and frailty populations including pregnant women, HIV patients, elderly peoples or children remaining largely inconclusive and requires further studies (Kahlig et al., 2021). There are only a few documented cases that implies that infection with *B. recurrentis* could have an adverse outcome for pregnant women (Kahlig et al., 2021).

At present, the gold standard for the diagnosis of LBRF is the microscopic visualization of spirochetes in Giemsa-stained thick blood smears during high spirochetemic episodes (<10^7^ bacteria/ml) of febrile patients (Cutler et al., 2017). Although conventional microscopy remains to be the gold standard, this method lacks sensitivity, in particular during afebrile episodes when the density of *B. recurrentis* in the blood is low (<10^4^ bacteria/ml) (Cutler et al., 2017). Enrichment of spirochetes by centrifugation followed by Giemsa staining increases the sensitivity and allows the detection of 10 spirochetes per ml of blood but visual inspection of blood smears is very time-consuming and requires qualified personnel experienced in microscopy Southern and Sanford, 1969; Larsson and Bergström, 2008). Cultivation of *B. recurrentis* in artificial medium is not a feasible diagnostic option given the lack of success to propagate spirochetes from freshly collected blood samples of febrile patients under sterile conditions coupled with the long cultivation time, and high costs for the growth medium (Fotso Fotso and Drancourt, 2015). Different suitable PCR-based protocols have already been developed as a point-of-care tool for the laboratory diagnosis of endemic tick-borne RF caused by *B. duttonii*, *B. hispanica*, and *B.  crocidurae* in Africa (Elbir et al., 2013). Despite the advantage of molecular detection, this methodology is cost-intensive and requires special equipment as well as well-trained technicians (Elbir et al., 2013; Fotso Fotso and Drancourt, 2015), hindering its use in developing countries. Reliable laboratory diagnosis is of utmost importance for appropriate treatment of patients with high fever because other concurrent diseases that cause high fever such as malaria, louse-borne typhus as well as other febrile illnesses such as leptospirosis, typhoid and hemorrhagic fever. Importantly, no commercial *in vitro* assay is available for the serodiagnosis of LBRF probably due to the difficulties to propagate *B.  recurrentis in vitro*, the lack of information regarding suitable antigens, and their potential cross-reactivities to antibodies elicited during infection with treponemes or other spirochetes (*Leptospira* spp., *Borrelia* spp.) (Southern and Sanford, 1969; Larsson et al., 2009; Kahlig et al., 2021). Previous studies identified glycerophosphodiester phosphodiesterase (GlpQ) of *B. recurrentis* and other tick-borne spirochetes as a promising antigen that elicits a strong immune response, possesses high sensitivity, and allows discrimination between RF and Lyme borreliosis in regions where both pathogens circulate (Schwan et al., 1996; Porcella et al., 2000). However, anti-IgG antibodies directed against recombinant GlpQ are often absent in most serum samples collected from patients infected with *B. recurrentis* at early time points (5 to 17 days) of infection (Porcella et al., 2000).

In the present study, we sought to develop and evaluate a point-of-care test for the serodiagnosis of LBRF by utilizing serum samples from clinical- and culture-confirmed LBRF patients collected in the 1990´s in Ethiopia and during the course of the refugee crisis in 2015 (Fekade et al., 1996; Cutler et al., 2010; Hoch et al., 2015; Seilmaier et al., 2016). For pre-screenings, IgM and IgG immunoreactivities of these serum samples were tested by conventional immunoblotting employing whole cell lysate of *B. recurrentis*. In addition, CihC and HcpA known to interact with the innate immune system (Grosskinsky et al., 2009; Grosskinsky et al., 2010), six as yet uncharacterized lipoproteins (ORF1 to ORF6) of *B. recurrentis* and GlpQ were initially examined for serological testing of IgM and IgG reactive antibodies. In summary, we provide evidence that the most promising target candidates are CihC and GlpQ which were selected to develop and evaluate immunoassays achieving a high sensitivity and specificity for IgM and IgG.

## Materials and methods

### Human serum samples

For the development of an *in vitro* immunoassay for the serodiagnosis of LBRF, we employed human serum samples primarily collected from patients with different zoonotic and spirochetal diseases. These include samples from confirmed patients who migrated from the Horn of Africa to Germany during the refugee crisis in 2015 (n=12) (Hoch et al., 2015; Seilmaier et al., 2016). Twenty sera from patients with clinically diagnosed LBRF and confirmed microscopically from Ethiopia were also included (Fekade et al., 1996; Cutler et al., 2010). Furthermore, we also utilized serum samples from patients clinically diagnosed with various zoonotic and spirochetal illnesses including Lyme borreliosis (n = 57), syphilis (n = 20), leptospirosis (n = 16), leishmaniasis (n = 11), and tuberculosis (n = 11), as well as sera from patients with rheumatoid arthritis (n = 10) (Kraiczy et al., 2007). Sera from patients collected in Sudan and clinically diagnosed with malaria, tuberculosis, and visceral leishmaniasis (n = 68) were assessed as well. Serum samples of healthy blood donors (BD) (n = 100) were used as negative controls and were collected between August 28, 2020, and August 31, 2020, by the German Red Cross Blood Donor Service Baden-Wuerttemberg-Hesse, Germany.

### Bacterial strains and culture conditions

*Borrelia recurrentis* strain A17 (human blood isolate, Addis Abeba, Ethiopia) (Cutler et al., 1994) was cultured at 33 °C until the mid-exponential phase (5 x 10^7^ spirochetes per ml) in a modified MKP medium (Margos et al., 2015) containing 50% human serum (TCS, Biosciences Ltd., Buckingham, UK) and 5.5% of BSA (Sigma-Aldrich, St. Louis, MO). Cultures of *Escherichia coli* BL21 (DE3) (New England Biolabs, Ipswich, MA) and *E. coli* M15 (Qiagen, Hilden, Germany) harboring expression vectors for the production of different His-tagged proteins were propagated routinely at 37°C in yeast tryptone broth supplemented with ampicillin (50 µg/ml).

### Preparation of whole-cell lysates

Bacterial cells grown to mid-exponential phase were centrifuged at 6,000 × g and washed four times with cold Dulbecco´s PBS^++^ containing MgCl_2_ and CaCl_2_ (Sigma-Aldrich, St. Louis, MO). The sedimented bacterial cells were resuspended in PBS^++^ and then lysed six times for 30 s by sonification employing a Branson sonifier 450 (Branson Ultrasonics, Danbury, CT).

### SDS-PAGE and western blot analyses for the detection of *anti-Borrelia recurrentis* antibodies in patient sera

To detect and evaluate the reactivity of anti-*Borrelia* antibodies in patient sera, we performed western blot analyses. For this purpose, whole-cell lysates (360 µg per gel) of *B. recurrentis* A17 were subjected to 10% Tris/Tricine SDS-PAGE under reducing conditions and transferred to nitrocellulose membrane by semi-dry blotting as previously described (Kraiczy et al., 2001). Briefly, the membrane was cut into 5 mm strips and nonspecific binding sites were blocked with 5% non-fat dry milk in TBS-T (50 mM Tris-HCl pH 7.4, 200 mM NaCl, 0.1% Tween 20) for 1 h at RT. Thereafter, each membrane was washed four times in TBS containing 0.1% Tween 20 and then incubated with LBRF patient sera diluted 1:100 in sample dilution buffer (NovaTec Immundiagnostica GmbH, Dietzenbach, Germany). Subsequently, each membrane was rinsed four times with TBS containing 0.2% Tween 20 and further incubated with horseradish peroxidase-conjugated anti-human IgM (NovaLisa, dilution 1:10,000) or a horseradish peroxidase-conjugated anti-human IgG antibody (NovaLisa, 1:96,000) for 30 minutes at RT. After four additional washing steps with TBS 0.2% Tween 20, the protein-antigen complexes were detected by applying 3,3',5,5'-tetramethylbenzidine as substrate. Each strip was digitalized by using a GS-900 image densitometer (Bio-Rad Laboratories, Hercules, CA) and signals were analyzed using the Image Lab software version 6.0.1 (Bio-Rad Laboratories).

### Generation of expression vectors and purification of His-tagged proteins

We amplified all genes of interest encoding for the borrelial proteins investigated in our study by conventional PCR using specific primers (**Supplementary Table 1**). Following digestion with the respective restriction enzymes, the DNA fragments were inserted into the MCS of the pQE-30 Xa expression vector (Qiagen, Hilden, Germany) to produce recombinant proteins harboring a hexahistidine tag at their N-terminus. Expression vectors encoding for borrelial lipoproteins ORF1, ORF2, ORF3, ORF4, ORF5, and ORF6 of *B. recurrentis* A17 were kindly provided by Dr. Reinhard Wallich, Institute of Immunology, University Heidelberg, Germany. Generation and transformation of the expression vectors containing the CihC and HcpA encoding genes of *B. recurrentis* A17 were described previously (Grosskinsky et al., 2009; Grosskinsky et al., 2010). To increase the yield and purity of CihC, the encoding gene was re-cloned into the pET-16b expression vector (Merck, Darmstadt, Germany) by using primers ChiC_Nde_FP and ChiC_Bam_RP (**Supplementary Table 1**). The resulting vector pET-CihC was then transformed into *E. coli* M15 according to the manufacturer’s instructions. Two CihC variants harboring either the N-terminal domain (ChiC-N) encompassing amino acids 20 to 194 or the C-terminal domain (CihC-C) with amino acids 195 to 356 were also generated by using specific forward and reverse primers for PCR amplification (**Supplementary Table 1**). The resulting DNA fragments were digested with *NdeI* and *BamHI* and the purified inserts were ligated into the MCS of the pET-16b expression vector. The ligation reactions were then transformed into *E. coli* BL21 (DE3) cells. In addition, the GlpQ encoding genes were PCR-amplified by using genomic DNA from *B. recurrentis* A17 as target and primers GlpQ BRE_242 Bam and GlpQ BRE_242 Sal (**Supplementary Table 1**). The amplified PCR product was inserted into the BamHI and SalI restriction sites of pQE-30 Xa and the ligation reactions transformed into *E. coli* BL21 (DE3) cells for the production of polyhistidine-tagged proteins. Plasmids isolated from the selected transformants were confirmed by restriction digestion following 1% agarose gel electrophoresis and by sequencing of the inserts (Eurofins Genomics Germany, Ebersberg, Germany). The herein applied expression and purification of recombinant proteins by Ni-metal affinity chromatography using the NEBExpress Ni resin (New England Biolabs, Ipswich, MA) have been described previously (Schmidt et al., 2021).

### Evaluation of a line blot immunoassay for the serodiagnosis of LBRF

We conducted the line blot immunoassays to identify promising candidate antigens for the *in vitro* diagnosis of LBRF. In contrast to conventional western blotting in which proteins are transferred to a solid membrane after separation through SDS-PAGE, the line blot is an immunoassay in which the purified antigens are directly sprayed on the membrane. One advantage of the line blot is that the purified proteins retain their native conformation. Purified antigens (for IgM, ChiC 50 µg/ml and GlpQ, 40 µg/ml; for IgG, ChiC 40 µg/ml and GlpQ, 30 µg/ml each) were printed in parallel onto nitrocellulose membranes (GE Healthcare, Chicago, IL) using a FrontLine HR Microliter Contact dispenser (BioDot, Irvine, CA) with a constant flow rate of 0.7 µl/cm. Upon drying, the membranes were cut into 3 mm strips and stored at 4 °C in sealable plastic bags. For the line blot immunoassay, each strip was incubated with the respective serum sample diluted 1:100 in 1 ml sample dilution buffer (NovaTec Immundiagnostica, Dietzenbach, Germany) for 1 h at RT, followed by three washing steps of 5 min at RT with NovaTec wash buffer. Thereafter, horseradish peroxidase-conjugated anti-human IgM or IgG antibodies (NovaLisa conjugate, NovaTec Immundiagnostica) were added and incubated for 30 min at RT. Finally, after three additional washing steps, the strips were developed with 3, 3', 5, 5'- tetramethylbenzidine (TMB) for 10 min, and the reactions were terminated by rinsing the strips several times with deionized water. Each membrane was digitalized by employing the GS-900 image densitometer (Bio-Rad) and the signal intensities were quantified by using the Image Lab software version 6.0.1 (Bio-Rad).

### Development and evaluation of a microtiter-based in vitro test (ELISA) for the diagnosis of LBRF

We developed an ELISA as an additional *in vitro* test for the diagnosis of LBRF. For this purpose, purified CihC, CihC-N, CihC-C, and GlpQ (1 ng/µl each), respectively were coated separately or in combination with GlpQ on a 96-well medium-bind microtiter plate (Greiner, Nürtingen, Germany) overnight at 4°C and afterward blocked with PBS containing BSA (0.45 %). The microtiter plates were air-dried and stored for several weeks at 4°C upon use. For each test, 100 µl of patient samples diluted 1:100 in sample dilution buffer (NovaTec Immundiagnostica GmbH) was added to the wells and incubated for 1 h at 37°C, respectively. The wells were then washed three times with NovaTec wash buffer and subsequently incubated with horseradish peroxidase-conjugated immunoglobulins (IgM, 1:50,000; IgG, 1:360,000) (NovaLisa conjugate, NovaTec Immundiagnostica GmbH) for an additional 30 min at RT. Subsequently, the wells were washed three times with NovaTec wash buffer and the protein complexes were detected by adding 100 µl TMB solution (NovaTec Immundiagnostica GmbH). The reactions were developed for 15 min at RT and terminated by adding 100 µl stop solution (NovaTec Immundiagnostica GmbH). The absorbance was measured at 450 nm and 620 nm as a reference using a PowerWave HT spectrophotometer (BioTek, Winooski, VT).

### Immunofluorescence microscopy

To confirm antigen reactivity of sera collected from patients with RF, we performed an immunofluorescence assay. Cells of *B. recurrentis* A17 grown to mid-exponential phase were harvested by centrifugation at 5,000 × g for 20 min and washed twice with GVB containing 0.5mM MgCl_2_ and 0.15 mM CaCl_2_ (GVB^++^) (ComTech, Tyler, TX). Afterward, the cell concentration was determined by dark-field microscopy using a Kova counting chamber (Hycor Biomedical, Garden Grove, CA). Spirochetes (3.6 × 10^4^) in 40 µl PBS^++^ containing 1% BSA were spotted onto microscope glass slides. These slides were air-dried overnight and subsequently fixed for 10 min with a 3% v/v glyoxal solution (Richter et al., 2018). After thorough washing glass slides were dried and incubated for 1 h with serum samples of LBRF patient sera (diluted 1:320 in PBS^++^ containing 1% BSA) at RT in a humidified chamber. The slides were then incubated for 1 h at RT with a ready-to-use fluorescein (FITC) conjugated goat anti-human IgG antibody solution (Euroimmun, Lübeck, Germany) followed by three washes with PBS^++^. Thereafter, spirochetal DNA was stained by adding 4′,6′-diamidino-2-phenylindole (DAPI) (10  μg/ml dissolved in PBS) for 10 min at 4°C. Following three additional washing steps with PBS^++^, the glass slides were sealed with Fluorescence Mounting Medium (Agilent Technologies, Santa Clara, CA) and a coverslip. Spirochetes were examined and digitalized at a magnification of ×1000 under an Axio Imager M2 fluorescence microscope (Zeiss, Oberkochen, Germany) equipped with a Spot RT3 camera (Visitron Systems, Puchheim, Germany).

### In-silico analysis

For in-silico analysis of CihC, the “Bepipred Linear Epitope Prediction 2.0” tool from IEDB Analysis Resource software was applied (tools.immuneepitope.org).

### Statistical analysis

To evaluate the performance of the line blot immunoassay and ELISA in discriminating between LBRF-positive and LBRF control sera, we conducted a receiver operating characteristic (ROC) analysis and calculated the area under the curve (AUC), the cutoff values and threshold, the sensitivity, as well as specificity. Thus, the sensitivity calculated means the percentage of true positive tests when using the LBRF sera, while the specificity is the percentage of true negative tests using the control sera. The statistical analyses were performed using GraphPad PRISM 8.0. (GraphPad Software, San Diego, Ca, USA).

## Results

### Assessment of antibody reactivities of sera from LBRF patients

**Figure 1:**
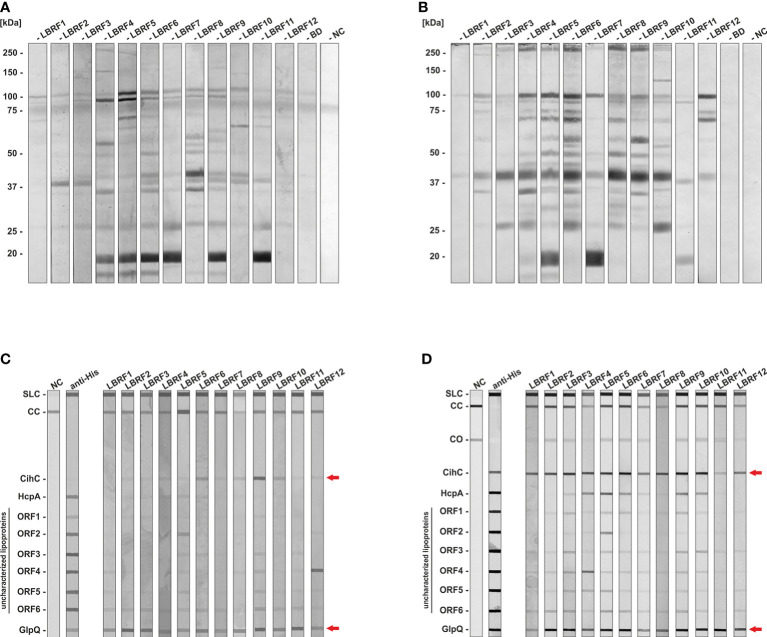
Immunoblot analysis to identify immunoreactive antigens of *B. recurrentis*. LBRF sera were assayed against whole cell antigens of *B. recurrentis* A17 by immunoblotting **(A, B)**. *Borrelia* antigens (360 µg) were separated and transferred to nitrocellulose and the membrane was cut into strips. LBRF sera diluted 1:100 were applied and IgM **(A)** and IgG **(B)** antibody responses were detected. LBRF, Louse-borne relapsing fever; NC, negative control containing serum-free dilution buffer; BD, blood donor. The mobilities of molecular mass standards in kDa are indicated on the left. LBRF sera were assayed against selected antigens by line blotting **(C, D)**. Purified antigens were printed on a membrane and IgM **(C)** and IgG responses were detected **(D)**. To verify that all antigens were properly printed, detection with an anti-His antibody (1:1000) was performed. Incubation with the secondary antibody only was used as a negative control (NC). Arrows indicate reactivities of patient sera to CihC and GlpQ. SLC, Sample loading control; CC, Conjugate control for IgM and IgG; CO, cut off control; ORF, Open reading frame.

Immunoreactivity of serum samples obtained from patients with clinically diagnosed and PCR and/or cultured confirmed LBRF (Hoch et al., 2015) was initially assessed by immunoblotting using whole-cell lysates from *B. recurrentis* strain A17. All serum samples tested showed a strong immunoreactivity to IgM or IgG with a robust IgM antibody response to an 18-kDa protein in six and a considerably stronger IgG response to a 41-kDa protein in seven out of twelve serum samples (**Figures 1A, B**). In addition, stronger immunoreactivities against multiple proteins of approximately 100-, 95-, 70-, 60-, 48- , 35-, and 27-kDa were found in several LBRF sera. The largest number of immunoreactive antigens was observed when IgG antibody responses were analyzed.

In order to confirm IgG reactivity of LBRF patient sera to native *B. recurrentis* antigens, immunofluorescence microscopy was conducted. Microscopic inspection revealed a strong staining of RF spirochetes for all patient sera analyzed whereas serum from a BD displayed no staining of spirochetes at all (**Supplementary Figure 1**). This finding indicates that the LBRF sera tested are valuable tools for the identification of serodiagnostic antigens.

### Verification of the IgM and IgG antibody reactivity to outer surface proteins of *Borrelia recurrentis*

Previously, we identified two outer surface lipoproteins, CihC and HcpA of *B. recurrentis* known to interact with components of the innate immune system, in particular complement C1 inhibitor, C4b binding protein, and Factor H (Grosskinsky et al., 2009; Grosskinsky et al., 2010; Röttgerding and Kraiczy, 2020). Due to their immunomodulatory function, we assumed that these particular lipoproteins might serve as potential serodiagnostic antigens as previously shown for the complement-inhibitory proteins CspA and CspZ of *Borrelia burgdorferi* (Rossmann et al., 2006; Kraiczy et al., 2007). These borrelial proteins and six additional HcpA homologous identified by bioinformatics were also included to test their immunoreactivity against LBRF sera. Furthermore, we also employed GlpQ of *B. recurrentis* A17 which has already been described as a valuable discriminatory antigen for the serodiagnosis of RF and Lyme borreliosis (Schwan et al., 1996; Porcella et al., 2000; Lopez et al., 2009; Lopez et al., 2010). Accordingly, purified antigens printed on nitrocellulose membranes were incubated with the LBRF patient sera, and signals obtained were digitally processed (**Figures 1C, D**). Concerning IgM antibody responses, GlpQ exhibited immunoreactivity to all patient sera. In addition, weaker reactivities to four out of 12 serum samples could be observed for CihC (**Figure 1C**). Similar results were observed when IgG antibody responses were analyzed employing GlpQ (**Figure 1D**). In contrast to IgM, all LBRF patient sera displayed IgG antibodies to CihC. Serum LBRF4, LBRF5, LBRF6, LBRF9, and LBRF10 had IgG antibodies to HcpA, and IgG responses to ORF2 and ORF4 were also detected for serum samples LBRF4 and LBRF5, respectively. None of the LBRF sera displayed IgM or IgG responses to the other HcpA orthologs. Therefore, CihC and GlpQ were selected and explored for all further immunoassays.

### Development and evaluation of a line blot immunoassay for the serodiagnosis of LBRF

**Figure 2:**
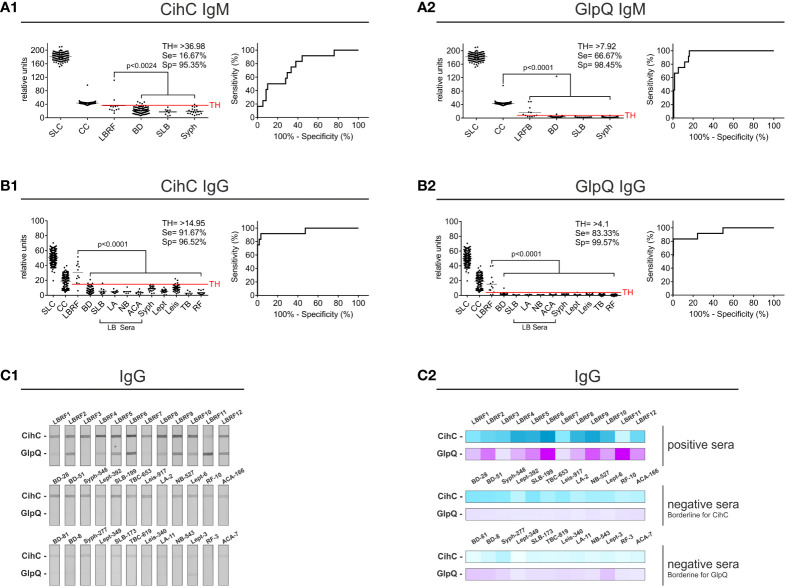
Evaluation of the IgM and IgG line blot immunoassay employing CihC and GlpQ. Membrane strips prepared with CihC and GlpQ were incubated with the LBRF patient sera and with different control sera. All strips were digitalized, and relative units were determined. The significance for the detection of the LBRF positive sera compared to the control sera is indicated (p-values). Sensitivity (Se) and specificity (Sp) were determined using the ROC curve. Values above the threshold (TH) were considered positive. **(A1, A2)** Results of the IgM line blot immunoassays with CihC and GlpQ. **(B1, B2)** Results of the IgG line blot immunoassays with CihC and GlpQ. **(C1, C2)** Scans of the IgG line blot immunoassays incubated with LBRF sera or incubated with selected control sera displaying borderline signals for CihC and GlpQ including a heat map of the signals shown. SLC, Sample Loading Control; CC, Conjugate Control for IgM and IgG; LBRF, louse-borne relapsing fever; BD, blood donor; SLB, serological-confirmed Lyme borreliosis; LA, Lyme arthritis; NB, neuroborreliosis; ACA, acrodermatitis chronica atrophicans; Syph, syphilis; Lept, leptospirosis; Leis, leishmaniasis; TB, tuberculosis; RF, rheumatoid arthritis; LB sera, Lyme borreliosis sera.

Having identified CihC and confirmed GlpQ as potential antigens for the serodiagnosis of LBRF, membranes containing both proteins were produced as described above. The membrane strips prepared were incubated with sera from LBRF patients and sera obtained from patients with a positive serology for Lyme borreliosis (SLB), syphilis, leptospirosis, visceral leishmaniasis, tuberculosis, and rheumatoid arthritis, as well as with sera from healthy BD (**Supplementary Table 2**). These latter serum samples were considered as control sera. IgM and IgG antibody reactivities obtained were densitometrically quantified and the signal intensities expressed as relative units were used to calculate sensitivity and specificity, and to generated the ROC curves (**Supplementary Table 3**). Concerning the IgM and IgG line blot immunoassay, all LBRF patient sera were considered positive (p<0.001) when compared with the BD sera (**Supplementary Figures 2A1, A2**) as well as the full control panel (**Figure 2A1**). IgM immunoreactivity against CihC, was less significant for the LBRF patient sera compared with the BD sera (p<0.045) exhibiting a sensitivity of 16.67% (**Supplementary Figure 2A1**). An identical sensitivity was calculated when the full control panel (BD, SLB, and syphilis) was included in the calculation (16.67%) (**Figure 2A1**). In comparison, the IgM immunoreactivity against GlpQ achieved a sensitivity of 66.67% and a specificity of 98% (BD sera only) and 98.45% (control panel included, **Figure 2A2**). The line blot immunoassay with CihC for IgG showed a sensitivity of 91.67% and a specificity of 97% compared with the BD sera (**Supplementary Figure 2B1**). Similar percentages were achieved (91.67% sensitivity and 96.52% specificity) with a TH of >14,95 (best trade-off between specificity and sensitivity) when all control sera were assessed (**Figure 2B1**). A ROC analysis for the IgM line blot immunoassay with CihC yielded an AUC of 0.9683 for the BD sera and an AUC of 0.9563 for the full control panel (**Supplementary Table 3**). While the GlpQ IgG line blot immunoassay yielded a sensitivity 83.33% with a specificity of 99.57% and the ROC analysis showed an AUC of 0.9175 compared with the BD sera and an AUC of 0.9373 for all controls (**Supplementary Figure 2B2** and **Figure 2B2**).

The lower sensitivity obtained using CihC in the IgM line blot immunoassay was attributed to a larger number of control sera displaying cross-reactivities to this particular antigen (**Figures 2C1, C2**, middle panel). Compared with CihC, most control panel serum did not show IgM immunoreactivity against GlpQ (**Figures 2C1, C2**, lower panel). These finding indicate that both antigens, CihC and GlpQ are promising candidates for the line blot immunoassay.

### Development and evaluation of an ELISA for the serodiagnosis of LBRF

**Figure 3:**
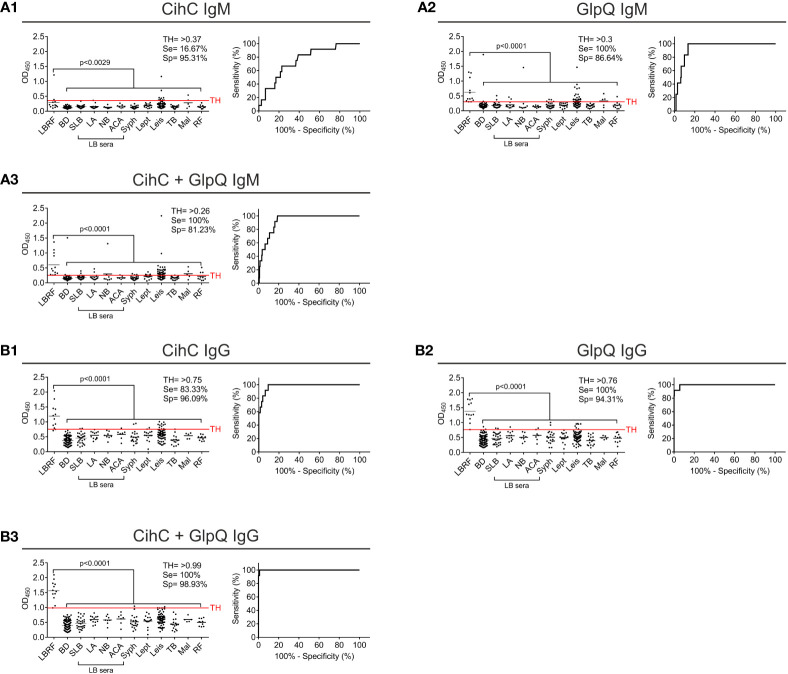
Evaluation of IgM and IgG ELISA employing CihC and GlpQ. Purified CihC and GlpQ were immobilized individually or in combination each at a concentration of 1 ng/µl. Microtiter plates were then incubated with different sera. Significant difference (LBRF positive sera compared to control sera) is shown. Specificities (Sp) and sensitivities (Se) are indicated. **(A)** Results of the IgM immunoreactivities to CihC, GlpQ or both antigens. **(B)** Results of the IgG immunoreactivities to CihC, GlpQ, or both antigens. Each circle represents one individual serum sample. LBRF, louse-borne relapsing fever; BD, blood donor; SLB, serological-confirmed Lyme borreliosis; LA, Lyme arthritis; NB, neuroborreliosis; ACA, acrodermatitis chronica atrophicans; Syph, syphilis; Lept, leptospirosis; Leis, leishmaniasis; TB, tuberculosis; Mal, malaria; RF, rheumatoid arthritis; LB sera, Lyme borreliosis sera.

Next, we sought to develop an ELISA as a standardized application in infection serology and to approve IgM and IgG antibody responses against CihC and GlpQ. As expected, IgM and IgG antibodies to both antigens were significantly detected in all LBRF sera investigated (**Figure 3**). Concerning IgM, the CihC ELISA showed a sensitivity of 66.67% and a specificity of 96% (BD sera only) (**Supplementary Figure 3A1**). When the full control panel was included, a sensitivity of 16,67% and a specificity of 95,31% was obtained (**Figure 3A1**). Concerning GlpQ, the sensitivity reached 100% and the specificity 98% with a TH of >0.3 (employing BD sera only) (**Supplementary Figure 3A2**). Utilizing all control sera for the calculation, the sensitivity was 100% and the specificity 86.64% (**Figure 3A2**). Similar results were obtained by combining both borrelial antigens. The IgM ELISA showed a sensitivity of 100% and a specificity of 98% when compared with the BD sera (**Supplementary Figure 3A3**). By considering the control sera into the calculation, the sensitivity was still 100% but the specificity slightly declined to 81.23 % (**Figure 3A3**).

Next, we assessed IgG immunoreactivity of all serum samples employing ELISA. By investigating CihC, a sensitivity of 100% and a specificity of 99% was achieved when compared with the BD sera (**Supplementary Figure 3B1**). When the control panel was included into the analyses, the sensitivity declined to 83.33% and the specificity to 96.09% (**Figure 3B1**). Using GlpQ, a sensitivity of 100% and a specificity of 98% could be calculated (BD sera only) (**Supplementary Figure 3B2**). The sensitivity (100%) was not affected but the specificity declined to 94.31% when the control panel was included (**Figure 3B2**). Finally, both antigens were simultaneously immobilized on microtiter plates and the respective sensitivities and specificities were calculated. By combining both antigens, the sensitivity and specificity were 100% (BD sera only) (**Supplementary Figure 3B3**). The specificity slightly decreased to 98.93% by a sensitivity of 100% when all control sera were assessed (**Figure 3B3** **)**.

### *In-silico* analysis of CihC

**Figure 4:**
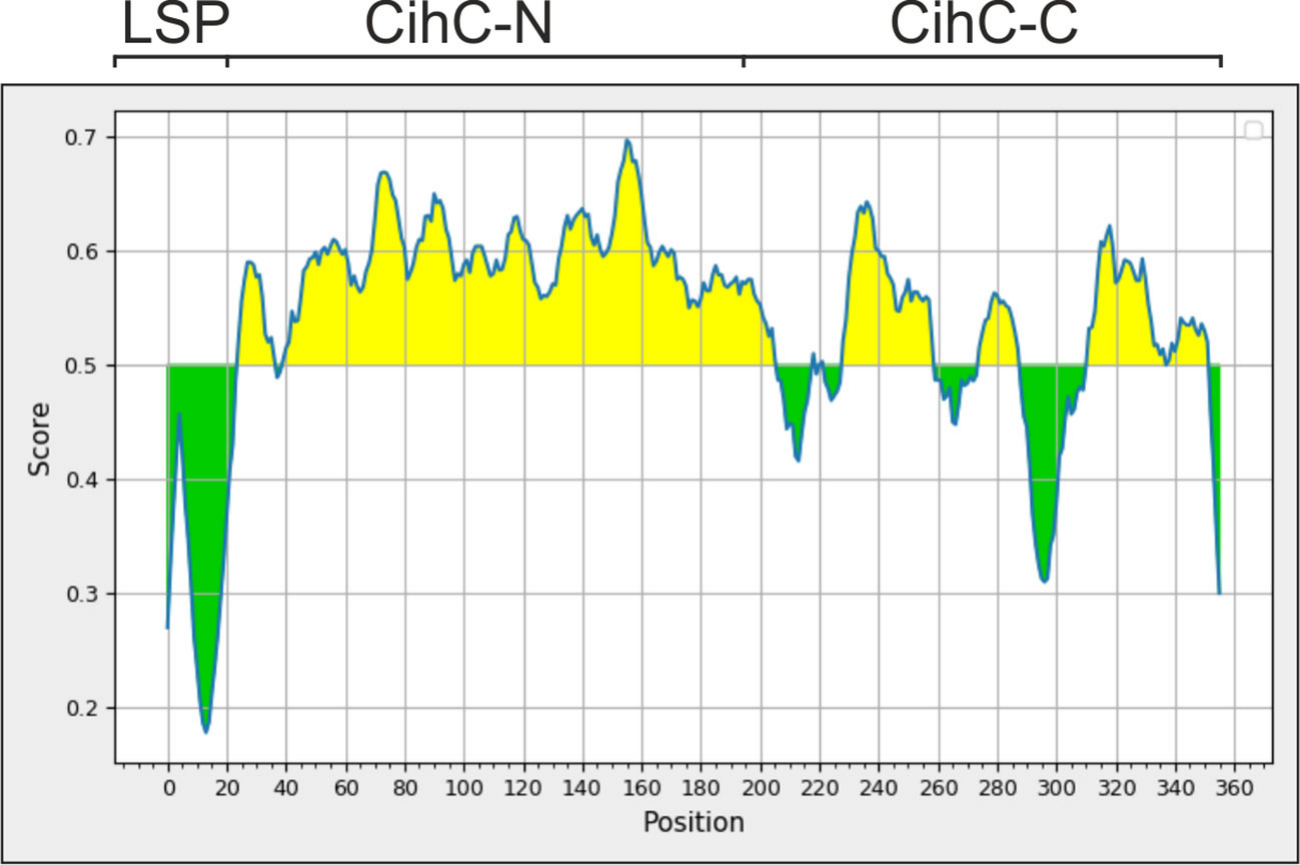
Determination of immunogenic regions within CihC by an in-silico analysis. For identifying immunogenic region, the “Bepipred Linear Epitope Prediction 2.0" was used resulting in the partitioning of CihC into a N-terminal CihC fragment (CihC-N) and a C-terminal fragment (CihC-C). LSP, Lipoprotein signal peptid.

Having demonstrated high specificity of ChiC for the detection of IgG responses in our LBRF serum samples, we sought to generated fragments displaying comparable immunoreactivity with the whole CihC protein. Due to technical hurdles, CihC could only be purified in low quantities independently of protocols tested, making this protein less attractive for a broader commercial application. To streamline the purification process, an *in-silico* analysis was conducted to identify the immunogenic region(s) within the CihC protein following generation of CihC fragments for upscaling the production process. By using BepiPred-2.0, a sequence-based B-cell epitope prediction tool, designed for the development of peptide-based vaccines, a high immunogenic region was identified to the N-terminus of CihC (**Figure 4**). Based on these data, we generated two CihC fragments comprising the N-terminal region (CihC-N) that consists of amino acids 20 to 194, and the C-terminal region (CihC-C) that encompasses amino acids 195 to 356. Compared to CihC, both fragments could be produced in *E. coli* in higher amounts and with an increased purity. Upon evaluation of both fragments with LBRF sera using line blot immunoassay, similar reactivity was observed for the CihC-N fragment, but the CihC-C fragment failed to show reactivity for IgM or showed a reduced reactivity for IgG (**Supplementary Figure 4**).

### Optimization of the line blot immunoassay using an N-terminal fragment of CihC

**Figure 5:**
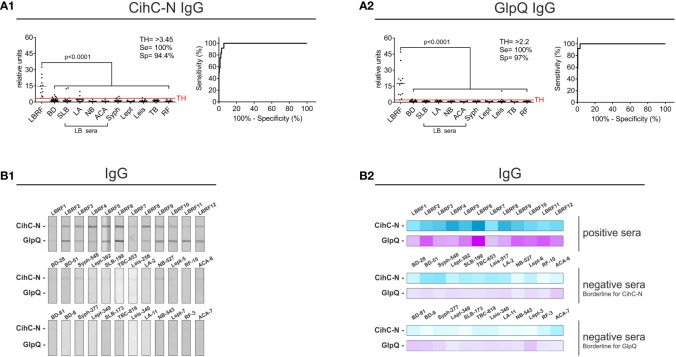
Improvement of the IgG line blot immunoassay employing the N-terminal CihC fragment. Membrane strips prepared with CihC-N and GlpQ were incubated with the LBRF positive and control sera. All strips were digitalized, and relative units were assessed. **(A)** Results of the IgG line blot immunoassays with CihC-N and GlpQ. **(B)** Scans of the LBRF patient sera and examples of control sera displaying the highest borderline signals to CihC-N and GlpQ including a heat map of the signals shown. LBRF, louse-borne relapsing fever; BD, blood donor; SLB, serological-confirmed Lyme borreliosis; LA, Lyme arthritis; NB, neuroborreliosis; ACA, acrodermatitis chronica atrophicans; Syph, syphilis; Lept, leptospirosis; Leis, leishmaniasis; TB, tuberculosis; RF, rheumatoid arthritis; LB sera, Lyme borreliosis sera.

To determine the immunoreactivity of the N-terminal ChiC-N fragment against LBRF sera, line blot immunoassays were conducted. Membrane strips containing ChiC-N and GlpQ were incubated with serum samples, and the IgM antibody responses were detected. Compared to the data obtained with whole ChiC protein, no significant increase in sensitivity and specificity was detected (data not shown). Concerning IgG responses, ChiC-N was immunoreactive to all LBRF sera conducted (p<0.001) as also observed for the whole ChiC protein (compare **Figures 2B1** with **5A1**). Also, the CihC-N IgG line blot immunoassay achieved a sensitivity of 100% and a specificity of 95% (BD sera only) (**Supplementary Figure 5**). By including the control panel for the calculation, the sensitivity was still 100% and the specificity slightly declined to 94.4% (**Figure 5A1**). In comparison, the GlpQ IgG line blot immunoassay showed a sensitivity of 100% and a specificity of 96% (BD sera only) (**Supplementary Figure 5**). When the full control panel was taken into consideration, a sensitivity of 100% and a specificity of 97% was obtained (**Figure 5A2**). Moreover, replacing ChiC by the N-terminal ChiC fragment could clearly reduce the cross-reactive signals and thereby enhance the sensitivity and specificity of the IgG line blot immunoassay (**Figures 5B1, B2**).

### Optimization of the ELISA using CihC-N as antigen

**Figure 6:**
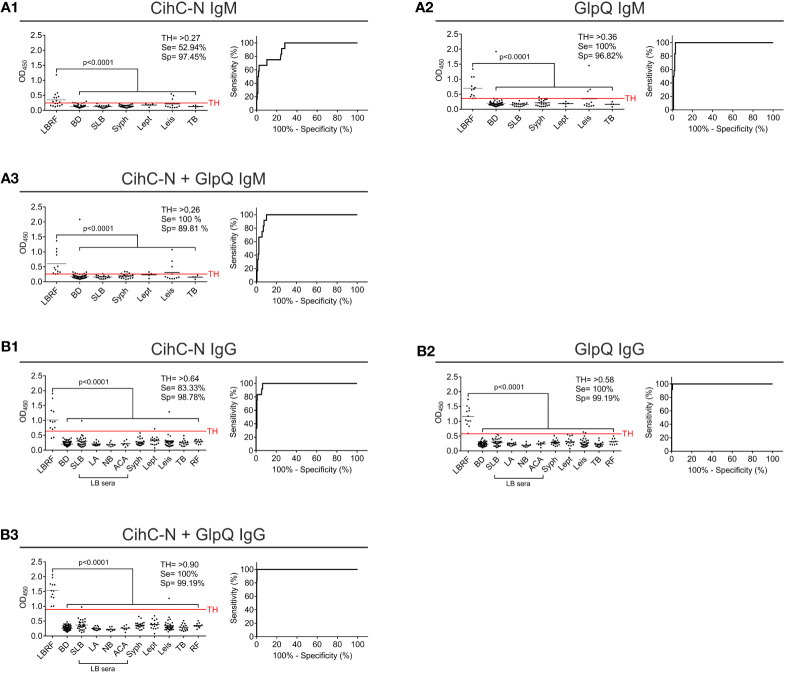
Re-evaluation of the IgG and IgM ELISA using CihC-N and GlpQ. Purified CihC-N and GlpQ were immobilized individually or in combination each at a concentration of 1 ng/µl. **(A1–A3)** Results of the IgM immunoreactivities to CihC-N, GlpQ or both antigens. **(B1–B3)** Results of the IgG immunoreactivities to CihC-N, GlpQ or both antigens. LBRF, louse-borne relapsing fever; BD, blood donor; SLB, serological-confirmed Lyme borreliosis; LA, Lyme arthritis; NB, neuroborreliosis; ACA, acrodermatitis chronica atrophicans; Syph, syphilis; Lept, leptospirosis; Leis, leishmaniasis; TB, tuberculosis; RF, rheumatoid arthritis; LB sera, Lyme borreliosis sera.

Having demonstrated the applicability of CihC-N as valuable antigen, further ELISA were performed. For this, we sought to include additional serum samples collected from LBRF patients from Ethiopia (see *Materials and methods*) (Fekade et al., 1996; Cutler et al., 2010). Initially, immunoreactivity of these sera was assessed by conventional western blotting employing whole cell lysates (**Supplementary Figure 6**). Sera exhibiting the strongest immunoreactivity (sample #48, #49, #56, #60, and #68) were selected for further analyses (**Supplementary Figure 6**). Concerning IgM responses, the CihC-N ELISA showed a sensitivity of 52.94% and a specificity of 95% in consideration with the BD sera only (**Supplementary Figure 7A1**) and a sensitivity of 52.94% and a specificity of 97.45% by considering the full control panel (**Figure 6A1**). The GlpQ IgM ELISA achieved a sensitivity of 100% and a specificity of 99% (BD sera only) (**Supplementary Figure 6A2**) and 96.82% (including all control sera), respectively (**Figure 6A2**). Microtiter plates prepared with both antigens achieved a sensitivity of 94.12% and a specificity of 95% (BD sera only) (**Supplementary Figure 6A3**). Taken all control serum samples into consideration, a sensitivity of 100% and a specificity of 89.81% could be calculated (**Figure 6A3**).

Measuring the IgG responses by ELISA with CihC-N as antigen, a sensitivity of 91.67% and a specificity of 100% (BD sera only) was determined (**Supplementary Figure 7B1**). The sensitivity (83.33%) and the specificity (98.78%) slightly declined when all control sera were included (**Figure 6B1**). As expected, the sensitivity and specificity (100%) did not change when IgG ELISA with GlpQ was investigated (compare **Figure 6B2** with **Supplementary Figure 7B2**). A sensitivity of 100 % and a specificity of 99.19% was achieved by incorporating all control sera (**Figure 6B3**). When both antigens were simultaneously immobilized, the sensitivity and specificity increased to 100% (BD sera only) (**Supplementary Figure 7B3**). Even if all LBRF control sera were included, a sensitivity of 100% and a specificity of 99.19% was obtained (**Figure 6B3**). These findings clearly indicate the potential of ChiC-N as a valuable serodiagnostic antigen for clinical practice.

## Discussion

LBRF is an archetypical and neglected vector-associated disease that resurged into focus during the 2015 refugee crisis (Hoch et al., 2015; Warrell, 2019; Kahlig et al., 2021). Even if there is currently no evidence for a new global spreading, a potential risk of the re-emergence of LBRF is, at any time, possible due to unnoticed endemic foci in refugee camps or in regions where the situation aggravates an already poor hygiene standard. Moreover, the inadequacy of our current diagnostics for LBRF highlighted by the 2015 crisis coupled with the urgent need for improved affordable diagnostics in endemic developing and in industrialized countries incentivized to develop standardized tests with high specificity and sensitivity for LBRF (Southern and Sanford, 1969; Larsson et al., 2009; Kahlig et al., 2021). Even though microscopic examination of blood smears is the gold standard, here we show that serological immunoassays are reliable for the diagnosis of LBRF as the evaluated tests described herein achieved high sensitivities and specificities for IgM and IgG.

Identification of superior immunoreactive antigens for the serodiagnosis of LBRF is of utmost importance for the development of a reliable immunoassay. Consequently, serum samples from clinical, culture- and laboratory-confirmed LBRF patients (Hoch et al., 2015), sera from patients with other spirochetal diseases such as LB, syphilis, and leptospirosis, sera from patients suffering from other vector-borne bacterial or parasitic diseases known to be endemic in East Africa such as visceral leishmaniasis, tuberculosis, malaria as well as serum samples from healthy BD were included as valuable controls (**Supplementary Table 2).**Conducting a serological pre-screening, most of the LBRF serum samples used herein examined show reactivity for IgM and IgG to a number of antigens ranged from <20 to 100 kDa (**Figure 1**). Our findings are consistent with data of a previous study investigating the reactivity of acute and convalescent phase serum samples of LBRF patients from Ethiopia to whole cell lysates of *B. recurrentis* (Porcella et al., 2000). In that study, indirect immunofluorescence assays were also applied for initial screening of anti-*Borrelia* antibodies, showing that the geometric mean titer for acute phase sera were 1:83 and for convalescent phase sera 1:575 which totally correspond with the dilution of 1:320 used to examined serological reactivity of our LBRF serum panel (**Supplementary Figure 1**). Concerning the antigens displaying a strong reactivity, seven of 12 LBRF sera (58 %) showed a response to a 20-kDa protein (IgM reactivity in six out of 12; IgG reactivity in three out of 12) (**Figure 1**) that most likely belong to the variable small protein (Vsp) family. In the same study, Porcella et al. (2000) reported that 67% of the convalescent-phase LBRF serum samples they analyzed had a strong reactivity to a putative 22-kDa Vsp protein of *B. recurrentis* which seems to be similar or identical with the immunoreactive 20-kDa protein detected herein. Additionally, we identified a further immunogenic 40-kDa protein that was recognized by 11 out of 12 LBRF sera. It was tempting to speculate that this particular protein could be either CihC (calculated MW of 40.5 kDa) or GlpQ (calculated MW of 38.1 kDa) both of which are highly similar in size and antigenicity. Of note, it cannot be completely excluded that these sera also contain antibodies displaying reactivity to flagellin, a borrelial protein that has a molecular mass of approximately 40 kDa. Comparative immunoblot analyses using whole cell lysates revealed that the signal obtained with a monoclonal anti-CihC Ab (Grosskinsky et al., 2010) recognizing the native CihC protein of *B. recurrentis* A17 correspond with the signals obtained with different LBRF sera (**Supplementary Figure 8**).

Among the borrelial lipoproteins examined, CihC was identified as the most promising target candidate revealing a strong IgG immunoreactivity among all LBRF sera tested and a weaker IgM reactivity in four serum samples (**Figure 2**). In addition, five LBRF sera had IgG reactivity to HcpA, and two sera recognize proteins ORF2 and ORF4. These findings suggested that proteins with immunomodulatory functions like CihC and HcpA are per se suitable antigens for serological immunoassays as previously discussed for the Factor H-binding protein FhbA of *B.  hermsii* as well as the Vmp proteins of *B. hermsii* and *B. miyamotoi* (Hovis et al., 2006; Lopez et al., 2009; Wagemakers et al., 2016; Jahfari et al., 2017; Koetsveld et al., 2018; Harris et al., 2019; Tokarz et al., 2020). The utility of GlpQ as a suitable antigen for the serodiagnosis of TBRF and *Borrelia miyamotoi* disease (BMD) has been previously reported (Schwan et al., 1996; Porcella et al., 2000; Nordstrand et al., 2007; Lopez et al., 2009; Jahfari et al., 2014; Koetsveld et al., 2018; Harris et al., 2019). Moreover, it has also been shown that GlpQ elicits a strong antibody response in patients infected with *B. recurrentis*, particularly with anti-GlpQ antibodies being detected in acute and in convalescent phase serum samples (Porcella et al., 2000) which is in agreement with the data present herein.

Our data support the concept of using two different antigens, CihC and GlpQ for serological immunoassays providing significantly improve specificity and sensitivity beyond that demonstrated for the diagnosis of BMD (Koetsveld et al., 2018; Harris et al., 2019). Furthermore, recent studies have shown that glycerophosphodiester phosphodiesterase (GlpQ) is a suitable candidate to potentially diagnose relapsing fever without causing cross-reactivity with serum samples from patients with LB (Porcella et al., 2000). Of note, LB sera from the panel employed did not display cross-reactivities to CihC as well.

Initial screenings employing line blot immunoassay revealed a quite low sensitivity of 16.67% but a high specificity of 95.35% for IgM using recombinant CihC (**Figure 2A1**). The same immunoassay achieved a sensitivity of 66.67% and specificity of 98.45% for GlpQ (**Figure 2A2**). As expected, high sensitivities and specificities were achieved by determining the IgG immunoreactivities (**Figure 2B1**). The higher signal-to-noise ratios obtained with the CihC-IgM immunoassay making the visual interpretation of the test results difficult (**Figure 2C**), which might prove challenging in resource poor regions that lack specific laboratory equipment used for data interpretation.

ELISA-based immunoassays are often used for pre-screening of patient samples to detect specific antibody responses. In addition, discrepancies between data obtained by ELISA and line blot immunoassays have previously been reported as well (Koetsveld et al., 2018). Although the IgM ELISA with the individual antigens exhibits a low sensitivity, in particular for CihC, a remarkable increase in sensitivity was obtained when both antigens were combined (**Figure 3A3**). Similarly, a high sensitivity and specificity for IgG were achieved for CihC in conjunction with GlpQ (**Figure 3B3**). Implementing of the N-terminal CihC fragment showing a strong immunoreactivity to certain LBRF sera (**Figure 5B**), however, did not impact the sensitivity of the IgM line blot immunoassay as expected but remarkable lower intensity signals could be achieved (**Figure 5B1**). Thus, a clearer discrimination between negative and positive test results without further digitalization leads to a reduce the hands-on time for thorough analyzation of the data when CihC-N was incorporated (**Figure 5B**).

Concerning the IgM ELISA, the sensitivity was improved of up to 30% using the ChiC-N fragment (**Figure 6A1** compared with **Figure 3A1**). However, only a few control immune sera from patients with other spirochetal diseases could be analyzed for the improved immunoassay with CihC-N that might impact the sensitivity of the test to some extent. Overall, CihC-N as antigens allows a better discrimination than CihC and the cut-off between positive and control sera was improved substantially. Compared to PCR-based tests, primarily established in specialized laboratories for species differentiation of RF borreliae (Elbir et al., 2013; Fotso Fotso and Drancourt, 2015), the overall high sensitivity and specificity making both immunoassays advantageous for point-of-care diagnostics. Especially the line blot immunoassay does not require cost-intensive laboratory equipment. A limitation of the IgM line blot immunoassay might be false-negative results in acute phase serum samples lacking appreciable anti-CihC and anti-GlpQ antibodies. Among the LBRF serum panel investigated, at least eight LBRF patients developed first symptoms shortly before or after their arrival in Bavaria and the other RF patients had only one and no recurrent fever episode reported suggesting blood collection at early stage of disease (Hoch et al., 2015; Kahlig et al., 2021). Nearly all serum samples were tested positive for GlpQ and/or CihC indicating that antibodies to both antigens developed at the acute phase of infection. However, the lower sensitivity of the IgM line blot immunoassay revealed the need for further optimization and evaluation of additional, more immunoreactive antigens such as Vmps. Nevertheless, the immunoassays developed are proposed to largely impact the current microbiological diagnostic of LBRF known to lack sensitivity, especially during the asymptomatic phase of bacterial relapses (Southern and Sanford, 1969; Wieser et al., 2016). Such immunoassays might be also of importance for the serodiagnosis of active cases in a human population repeatedly exposed to LBRF and where the detection of circulating antibodies would be suspected.

A well-known obstacle of all serological assays are cross-reactive antibodies in serum samples from patients with other spirochetal infections that often leads to false-positive results. BLAST searches revealed that GlpQ encoding genes are present in diverse human pathogenic bacteria including *Escherichia coli*, *Haemophilus influenzae*, *Pseudomonas aeruginosa*, *Salmonella* spp.*, Klebsiella* spp., and *Yersinia* spp. as well as several other human pathogenic species while homologs are absent in LD spirochetes. In agreement with previous reports (Schwan et al., 1996; Porcella et al., 2000; Krause et al., 2013; Jahfari et al., 2014; Krause et al., 2014; Molloy et al., 2015; Jahfari et al., 2017), we could not detect cross-reactive antibodies to GlpQ in the serum panels investigated, most likely due to the low sequence identity of the homolog proteins (below 50%). In contrast, GlpQ is highly conserved among the TBRF borreliae *B. hermsii*, *B. turicatae*, *B. parkeri*, *B. coriaceae* as well as *B. duttonii* sharing sequence identities of 82 to 99,4 % (Porcella et al., 2000; Elbir et al., 2013). As Lyme disease spirochetes lacking the GlpQ encoding gene, this protein was considered as a suitable target candidate for the serodiagnosis of TBRF, especially in regions where certain vectors carrying the respective pathogens circulate. Thus, we would expect detection of cross-reacting anti-GlpQ antibodies in sera from patients infected with *B. duttonii* or *B. crocidurae*, however, we were unable to analyze serum samples collected from TBRF patients.

Bioinformatic analyses utilizing BLASTp identified a CihC homologous protein in *B. duttonii* sharing a sequence identity of 89.2 % whereby no homologs could be detected in *B. hermsii*, *B. turicatae*, *B. parkeri*, and *B. crocidurae* suggesting that CihC is unique to *B. recurrentis* and *B. duttonii*. Similar to GlpQ, CihC cross-reactive antibodies could be expected in sera of *B. duttonii*-infected patients but should allow discrimination between TBRF caused by other RF spirochetes as well as BMD and Lyme borreliosis. It is well-known that serology lacks sufficient sensitivity to distinguish infections caused by RF borreliae in endemic region in Northern and Eastern Africa (Fotso Fotso and Drancourt, 2015; Kahlig et al., 2021). From clinical perspectives, differentiation between TBRF and LBRF would be a matter of scientific interest to collect epidemiological data but have no impact on the patient management and treatment (Cutler, 2010).

In conclusion, we developed and evaluated two immunoassays with high sensitivity and specificity which, according to our data, are reliable tools for the serodiagnosis of LBRF. Collectively, CihC and GlpQ were identified as promising target candidates for the detection of IgM and IgG responses in sera obtained from patients with LBRF. Optimization of our immunoassay by utilizing a N-terminal CihC fragment increases the overall sensitivity of this test system. Both immunoassays allow a more stringent and rapid diagnosis of patients with fever of unknown origin and may serve as reliable point-of-care diagnostic, especially in rural hospitals. In addition, the time point at which the presence of IgG antibodies against LBRF can be detected in patients after primary infection, as well as cross-reactive antibodies in TBRF patient samples, is a matter of further surveys. Once these data are available, these immunoassays might become important diagnostic tools in endemic regions for the early detection of LBRF, appropriate care of patients, and prevention of epidemic outbreaks.

## Data availability statement

The raw data supporting the conclusions of this article will be made available by the authors, without undue reservation.

## Ethics statement

The collection of blood samples from patients with the diagnosis of suspected Lyme borreliosis syphilis, leptospirosis, tuberculosis, and rheumatoid arthritis and the consent documents were approved by the ethics committee at the University Hospital of Frankfurt (control numbers 160/10, 222/14, and 423/11). Concerning serum samples collected from LBRF patients in Germany, ethical approval was not required because all samples used were remnants from routine diagnostics in the context of secondary use of biological material and without the possibility of inferring patient data. The collection of serum samples obtained from patients from Ethiopia was approved by the research ethics committee of the Faculty of Medicine, Addis Ababa University. Sera collected from African patients with the diagnosis of visceral leishmaniasis, malaria, or tuberculosis were collected in the rural hospital of Doka, Eastern State of Sudan. This study was approved by the ethical review committee of the Federal Ministry of Health in Sudan and all patients have given consent for participation in this study. All sera used in this study were anonymized. The patients/participants provided their written informed consent to participate in this study.

## Author contributions

Designed research: FR and PK. Performed research: FR, JN, and PK. Contributed new reagents and analytic tools: ES, AL, RM, US, SC, SB, VK, and VF. Analyzed data: FR and PK. Drafted and revised the paper: FR, PK, and SC. All authors contributed to the article and approved the submitted version.

## Funding

This work was supported by the LOEWE Center DRUID (Novel Drug Targets against Poverty-Related and Neglected Tropical Infectious Diseases), projects C2 (VAJK), C3 (PK), and C4 (US).

## Acknowledgments

The authors gratefully acknowledge the skillful and excellent technical assistance of Heike Podlich and Martyna Olesiuk. We are also indebted to Prof. Dr. Reinhard Wallich (emer.), Institute of Immunology, University Hospital of Heidelberg, Germany who kindly provided vectors. This work forms part of the doctoral thesis of FR.

## Conflict of interest

ES and AL are employees of NovaTec Immundiagnostica GmbH.

All authors declare that the research was conducted in the absence of any commercial or financial relationships that could be construed as a potential conflict of interest.

## Publisher’s note

All claims expressed in this article are solely those of the authors and do not necessarily represent those of their affiliated organizations, or those of the publisher, the editors and the reviewers. Any product that may be evaluated in this article, or claim that may be made by its manufacturer, is not guaranteed or endorsed by the publisher.
