# Supplementary material for: Novel approaches for the serodiagnosis of louse-borne relapsing fever
Source: Front Cell Infect Microbiol. 2022 Sep 20;12:983770. doi: 10.3389/fcimb.2022.983770 (PMC9530196; doi:10.3389/fcimb.2022.983770)
Supplement: Supplementary file 1 [file DataSheet_1.pdf]

Supplementary Table 1. Oligonucleotides used in this study

Oligonucleotide	Sequence (5’-3’)[a]	Use in this work
GlpQ BRE_242 Bam	GAGGTTAAATTAATGGGATCCAAATTAATAATG	PCR amplification
GlpQ BRE_242 Sal	CATAAATGAGATAGTCGACTTATTAAAATTATG	PCR amplification
ChiC-194 Stop Bam_RP	CTATCTAATTTATCATTGGATCCTTACTCCGGACTATCTTCTTCTTC	PCR amplification and generation of the CihC-N fragment
ChiC_Nde_FP	GGCCTTATAATGGAACTCATATGGAATATAATG	PCR amplification and generation of the CihC-C fragment
ChiC_Bam_RP	GGCTGCAGGTCGGATCCCGACTACTTTACATAC	PCR amplification and generation of the CihC-C fragment
M13 for	GTAAAACGACGCCCAGT	PCR amplification and sequencing
M13 rev	CAGGAAACAGCTATGAC	PCR amplification and sequencing
T7 Term_fwd	TATGCTAGTTATTGCTCAG	PCR amplification and sequencing
T7 Prom_fwd	TAATACGACTCACTATAGGG	PCR amplification and sequencing
pQE-FP-30	TTGCTTTGTGAGCGGATAAC	Sequencing
pQE-RP	CTGAGGTCATTACTGGATCTATC	Sequencing

[a], Sequences of specific restriction endonuclease recognition sites are underlined
