# Supplementary material for: Novel approaches for the serodiagnosis of louse-borne relapsing fever
Source: Front Cell Infect Microbiol. 2022 Sep 20;12:983770. doi: 10.3389/fcimb.2022.983770 (PMC9530196; doi:10.3389/fcimb.2022.983770)
Supplement: Supplementary file 2 [file DataSheet_2.pdf]

Supplementary Table 2. Serum samples used in the study

		Number of patient sera analyzed												
**Method**	**Antigen**	**LBRF**	**BD**	**SLB**	**LA**	**NB**	**ACA**	**Syph**	**Lept**	**Leis**	**TB**	**Mal**	**RF**	**Total**
Line blot (IgM)	CihC	12	100	9	n.d.	n.d.	n.d.	20	n.d.	n.d.	n.d.	n.d.	n.d.	**141**
	GlpQ	12	100	9	n.d.	n.d.	n.d.	20	n.d.	n.d.	n.d.	n.d.	n.d.	**141**
Line blot (IgG)	CihC	12	100	9	10	5	7	20	16	48	5	n.d.	10	**242**
	GlpQ	12	100	9	10	5	7	20	16	48	5	n.d.	10	**242**
ELISA (IgM)	CihC	12	100	29	12	8	7	20	15	55	16	5	10	**289**
	GlpQ	12	100	29	12	8	7	20	15	55	16	5	10	**289**
	CihC + GlpQ	12	100	29	12	8	7	20	15	55	16	5	10	**289**
ELISA (IgG)	CihC	12	100	29	12	8	7	20	15	69	16	5	10	**303**
	GlpQ	12	100	29	12	8	7	20	15	69	16	5	10	**303**
	CihC + GlpQ	12	100	29	12	8	7	20	15	69	16	5	10	**303**
Line blot (IgG)	CihC-N	12	100	29	12	8	6	20	9	28	11	n.d.	10	**245**
	GlpQ	12	100	29	12	8	6	20	9	28	11	n.d.	10	**245**
ELISA (IgM)	CihC-N	17	100	17	n.d.	n.d.	n.d.	20	5	11	4	n.d.	n.d.	**169**
	GlpQ	17	100	17	n.d.	n.d.	n.d.	20	5	11	4	n.d.	n.d.	**169**
	CihC-N + GlpQ	17	100	17	n.d.	n.d.	n.d.	20	5	11	4	n.d.	n.d.	**169**
ELISA (IgG)	CihC-N	12	100	30	12	8	7	20	15	29	15	n.d.	10	**258**
	GlpQ	12	100	30	12	8	7	20	15	29	15	n.d.	10	**258**
	CihC-N + GlpQ	12	100	30	12	8	7	20	15	29	15	n.d.	10	**258**

n.d.. not done; LBRF, louse-borne relapsing fever; BD, blood donor; SLB, serological-confirmed Lyme borreliosis; LA, Lyme arthritis; NB, neuroborreliosis; ACA, acrodermatitis chronica atrophicans; Syph, syphilis; Lept, leptospirosis; Leis, leishmaniasis; TB, tuberculosis; Mal, malaria; RA, rheumatoid factor
