# Supplementary material for: Novel approaches for the serodiagnosis of louse-borne relapsing fever
Source: Front Cell Infect Microbiol. 2022 Sep 20;12:983770. doi: 10.3389/fcimb.2022.983770 (PMC9530196; doi:10.3389/fcimb.2022.983770)
Supplement: Supplementary file 3 [file DataSheet_3.pdf]

Supplementary Table 3. Summary of test results

		Blood donor sera					Full control panel				
**Method**	**Antigen**	**Threshold**	**Sensitivity [%]**	**Specificity [%]**	**AUC**	**95 % CI**	**Threshold**	**Sensitivity [%]**	**Specificity [%]**	**AUC**	**95 % CI**
Line blot (IgM)	CihC	>36,98	16,67	95	0,7517	0,6136-0,8898	>36,98	16.67	95,35	0,7655	0,6351-0,8959
	GlpQ	>7,83	66,67	98	0,9508	0,9083-0,9934	>7,92	66.67	98,45	0,9522	0,9114-0,993
Line blot (IgG)	CihC	14,88	91,67	97	0,9683	0,9141-1,023	>14,95	91.67	96,52	0,9563	0,882-1,03
	GlpQ	3,86	83,33	99	0,9175	0,8095-1,025	>4,1	83.33	99.57	0,9373	0,8536-1,021
ELISA (IgM)	CihC	>0,19	66,67	96	0,9033	0,8108-0,9958	>0,37	16.67	95.31	0,7541	0,6275-0,8806
	GlpQ	>0,3	100	98	0,9883	0,9684-1,008	>0,3	100	86.64	0,9371	0,9041-0,9702
	CihC + GlpQ	>0,25	100	98	0,9883	0,9684-1,008	>0,26	100	81.23	0,927	0,8852-0,9689
ELISA (IgG)	CihC	>0,7	100	99	0,9992	0,9964-1,002	>0,75	83,33	96.09	0,9801	0,961-0,9993
	GlpQ	>0,76	100	98	0,9983	0,9939-1,003	>0,76	100	94.31	0,995	0,9855-1,004
	CihC + GlpQ	>0,9	100	100	1	1-1	>0,99	100	98.93	0,991	0,9969-1,001
Line blot (IgG)	CihC-N	>3,24	100	95	0,9933	0,9822-1,004	>3,45	100	94.42	0,9866	0,9762-1,001
	GlpQ	>2,06	100	96	0,9967	0,989-1,004	>2,2	100	97	0,9961	0,9897-1,002
ELISA (IgM)	CihC-N	>0,24	52,94	95	0,9238	0,857-0,9907	>0,27	52,94	97,45	0,891	0,9144-0,9675
	GlpQ	>0,36	100	99	0,99	0,9705-1,01	>0,36	100	96,82	0,9816	0,9624-1,001
	CihC-N + GlpQ	>0,27	94,12	95	0,9824	0,9602-1,005	>0,26	100	89.81	0,97	0,9462-0,9938
ELISA (IgG)	CihC-N	>0,45	91,67	100	0,9983	0,9939-1,003	>0,64	83.33	98.78	0,9856	0,9705-1,001
	GlpQ	>0,52	100	100	1	1-1	>0,58	100	99.19	0,9993	0,9975-1,001
	CihC-N + GlpQ	>0,75	100	100	1	1-1	>0,90	100	99.19	0,9993	0,9975-1,001

AUC, area under the curve; CI, confidence interval
