# Supplementary material for: Novel approaches for the serodiagnosis of louse-borne relapsing fever
Source: Front Cell Infect Microbiol. 2022 Sep 20;12:983770. doi: 10.3389/fcimb.2022.983770 (PMC9530196; doi:10.3389/fcimb.2022.983770)
Supplement: Supplementary file 4 [file DataSheet_4.pdf]

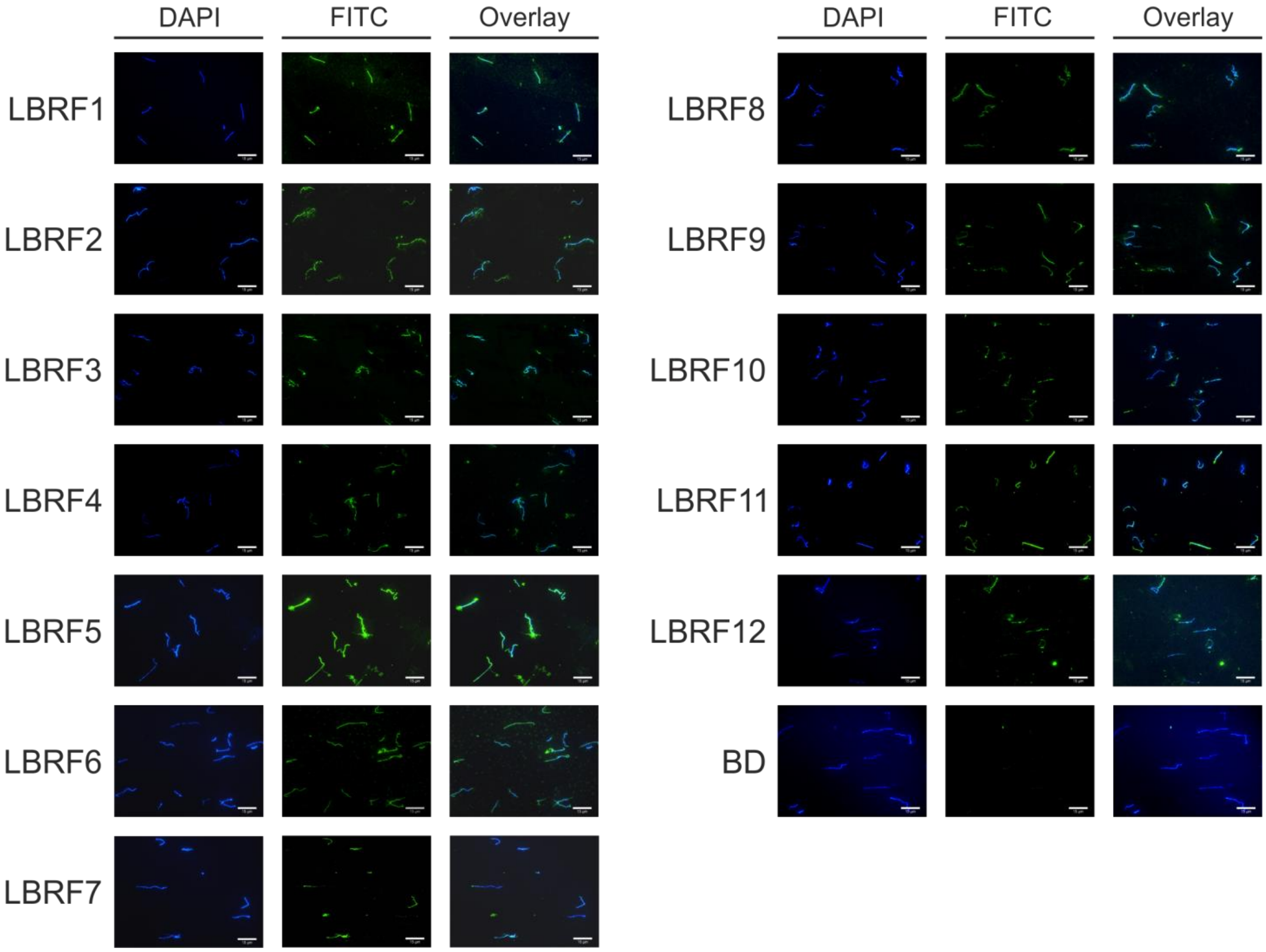

Supplementary figure 1. Assessment of the antibody reactivity of LBRF serum samples using immunofluorescence microscopy. *B. recurrentis* A17 (3.6 x 10^4 cells) were fixed onto glass slides and incubated with LBRF patient sera (dilution 1:320). Binding of antibodies were detected with a FITC-conjugated anti-IgG antibody (Euroimmun AG, Lübeck, Germany) (green) and spirochetal DNA (blue) was stained by employing the DNA-binding dye DAPI. The spirochetes were observed at a magnification of x 1000 and the data were recorded with an Axio Imager M2 fluorescence microscope (Zeiss) equipped with a Spot RT3 camera (Visitron Systems). All scale bars are equal to 15 µm., LBRF, louse-borne relapsing fever; BD, blood donor
