# Supplementary material for: Novel approaches for the serodiagnosis of louse-borne relapsing fever
Source: Front Cell Infect Microbiol. 2022 Sep 20;12:983770. doi: 10.3389/fcimb.2022.983770 (PMC9530196; doi:10.3389/fcimb.2022.983770)
Supplement: Supplementary file 5 [file DataSheet_5.pdf]

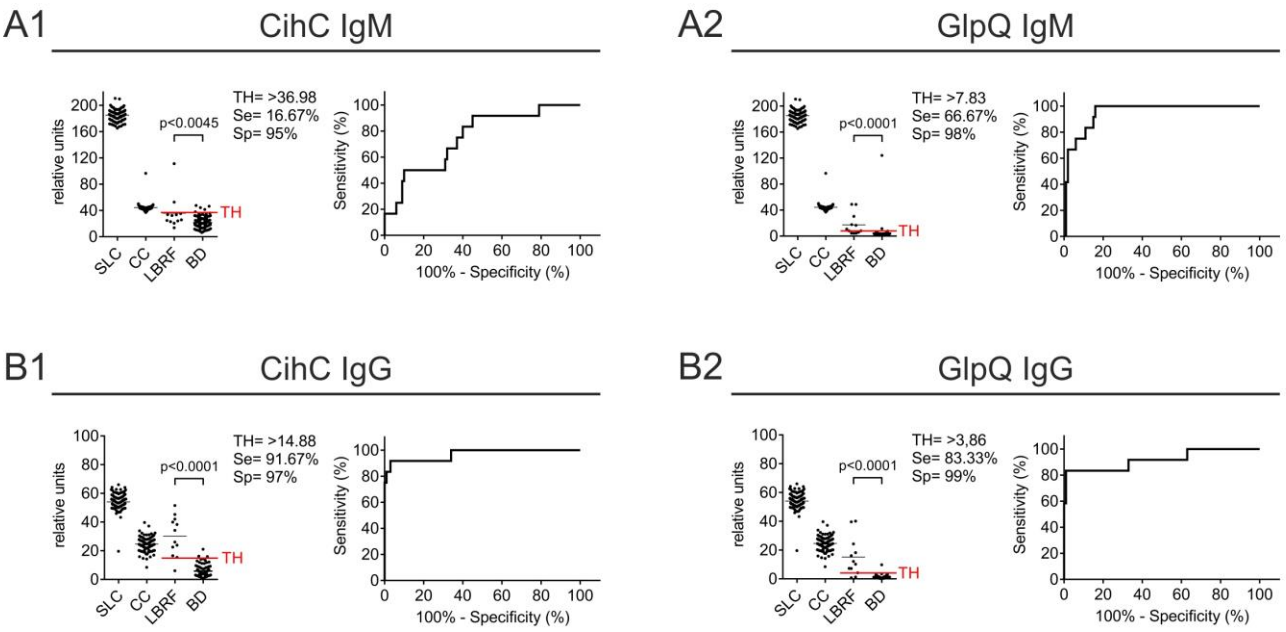

Supplementary figure 2. Evaluation of the IgM and IgG line blot immunoassay employing CihC and GlpQ and blood donor serum samples. Membrane strips prepared with CihC and GlpQ were incubated with the LBRF patient sera and with blood donor serum samples. All strips were digitalized, and relative units were determined. The significance for the detection of the LBRF positive sera compared to the control sera is indicated (p-values). Sensitivity (Se) and specificity (Sp) were determined using the ROC curve. Values above the threshold (TH) were considered positive. **(A)** Results of the IgM line blot immunoassays with CihC and GlpQ. **(B)** Results of the IgG line blot immunoassays with CihC and GlpQ. SLC, Sample Loading Control; CC, Conjugate Control for IgM and IgG; LBRF, louse-borne relapsing fever; BD, blood donor.
