# Supplementary material for: Novel approaches for the serodiagnosis of louse-borne relapsing fever
Source: Front Cell Infect Microbiol. 2022 Sep 20;12:983770. doi: 10.3389/fcimb.2022.983770 (PMC9530196; doi:10.3389/fcimb.2022.983770)
Supplement: Supplementary file 6 [file DataSheet_6.pdf]

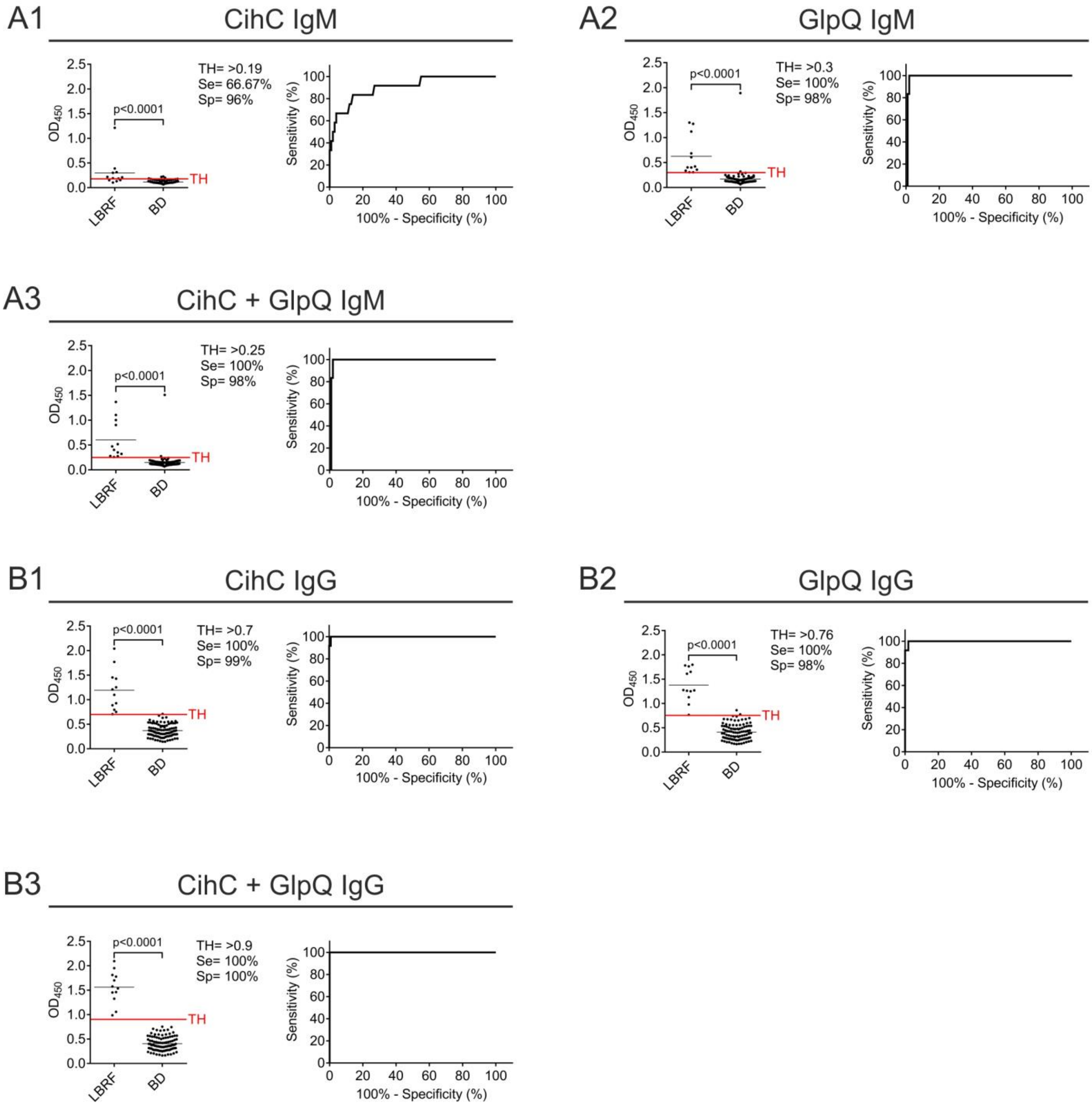

Supplementary figure 3. Evaluation of IgM and IgG ELISA employing CihC and GlpQ. Purified CihC and GlpQ were immobilized individually or in combination each at a concentration of 1 ng/µl. Microtiter plates were then incubated with different sera. Significant difference (LBRF positive sera compared to blood donor serum samples) is shown. Specificities (Sp) and sensitivities (Se) are indicated. **(A)** Results of the IgM immunoreactivities to CihC, GlpQ or both antigens. **(B)** Results of the IgG immunoreactivities to CihC, GlpQ**,** or both antigens. Each circle represents one individual serum sample. LBRF, louse-borne relapsing fever; BD, blood donor.
