# Supplementary material for: Novel approaches for the serodiagnosis of louse-borne relapsing fever
Source: Front Cell Infect Microbiol. 2022 Sep 20;12:983770. doi: 10.3389/fcimb.2022.983770 (PMC9530196; doi:10.3389/fcimb.2022.983770)
Supplement: Supplementary file 7 [file DataSheet_7.pdf]

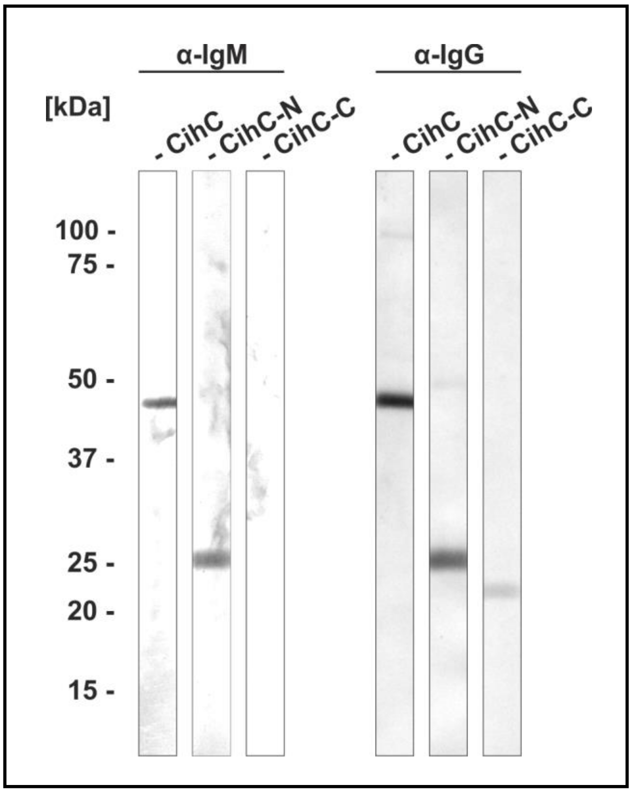

Supplementary figure 4. Determination of CihC in *B. recurrentis* A17 and detection of IgM and IgG responses to CihC. Purified His-tagged ChiC, His-tagged ChiC-N, and His-tagged CihC-C (500 ng each) were separated by a Tris/Tricine SDS-PAGE and transferred to a nitrocellulose membrane. The membrane was cut into strips and IgM and IgG antibody responses were detected using LBRF serum sample LBRF9 as an example. The mobilities of molecular mass standards in kDa are indicated on the left.
