# Supplementary material for: Novel approaches for the serodiagnosis of louse-borne relapsing fever
Source: Front Cell Infect Microbiol. 2022 Sep 20;12:983770. doi: 10.3389/fcimb.2022.983770 (PMC9530196; doi:10.3389/fcimb.2022.983770)
Supplement: Supplementary file 8 [file DataSheet_8.pdf]

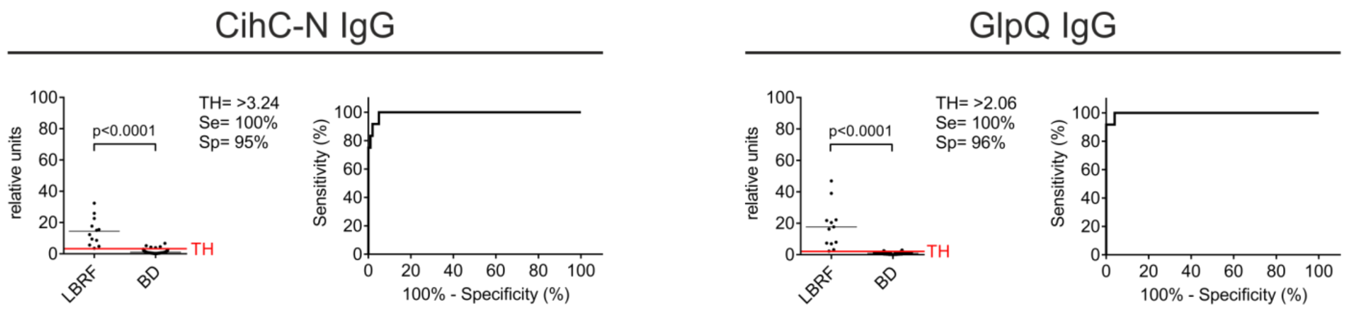


**Supplementary figure 5. Improvement of the IgG line blot immunoassay employing the N-terminal CihC fragment.** Membrane strips prepared with CihC-N and GlpQ were incubated with the LBRF positive and control sera. All strips were digitalized, and relative units were assessed. Significant difference (LBRF positive sere compared to blood donor serum samples) is shown above each graph. Specificities (Sp) and sensitivities (Se) as obtained by ROC curve analyses are indicated. Each sample with the relative unit value above the threshold (TH) was considered positive. Results of the IgG line blot immunoassays with CihC-N and GlpQ. LBRF, louse-borne relapsing fever; BD, blood donor.
