# Supplementary material for: Novel approaches for the serodiagnosis of louse-borne relapsing fever
Source: Front Cell Infect Microbiol. 2022 Sep 20;12:983770. doi: 10.3389/fcimb.2022.983770 (PMC9530196; doi:10.3389/fcimb.2022.983770)
Supplement: Supplementary file 9 [file DataSheet_9.pdf]

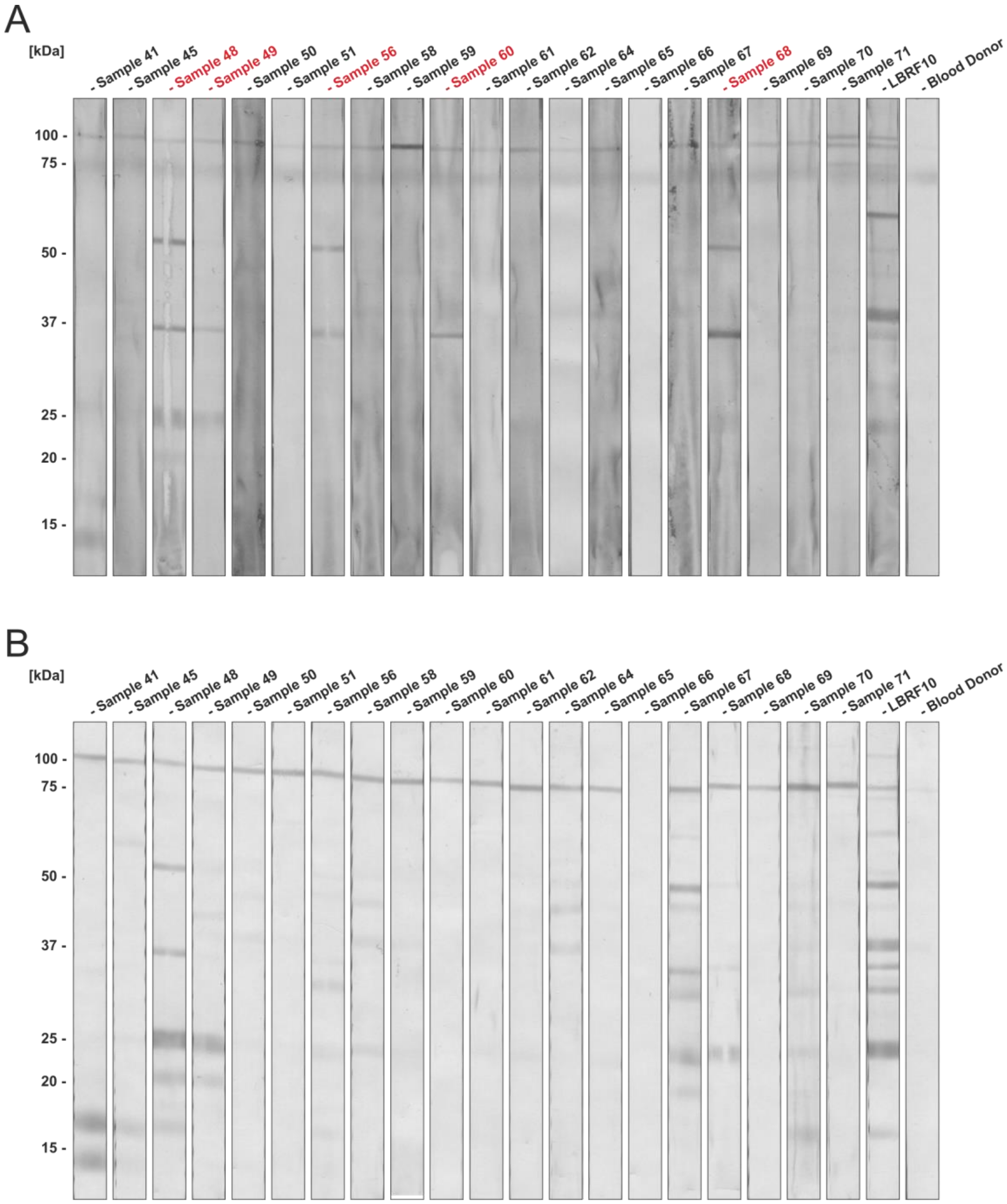

Supplementary figure 6. Immunoblot analysis to identify immunoreactive antigens of *B. recurrentis*. Additional LBRF sera were assayed against whole cell antigens of *B. recurrentis* A17 by immunoblotting (**A** and **B**). *Borrelia* antigens (360 µg) separated by Tris/Tricine SDS-PAGE were transferred to nitrocellulose and the membrane was cut into strips. LBRF sera diluted 1:100 were applied and IgM **(A)** and IgG **(B)** antibody responses were detected. Serum samples marked in red were used for ELISA. The mobilities of molecular mass standards in kDa are indicated on the left. LBRF, Louse-borne relapsing fever.
