# Supplementary material for: Novel approaches for the serodiagnosis of louse-borne relapsing fever
Source: Front Cell Infect Microbiol. 2022 Sep 20;12:983770. doi: 10.3389/fcimb.2022.983770 (PMC9530196; doi:10.3389/fcimb.2022.983770)
Supplement: Supplementary file 10 [file DataSheet_11.pdf]

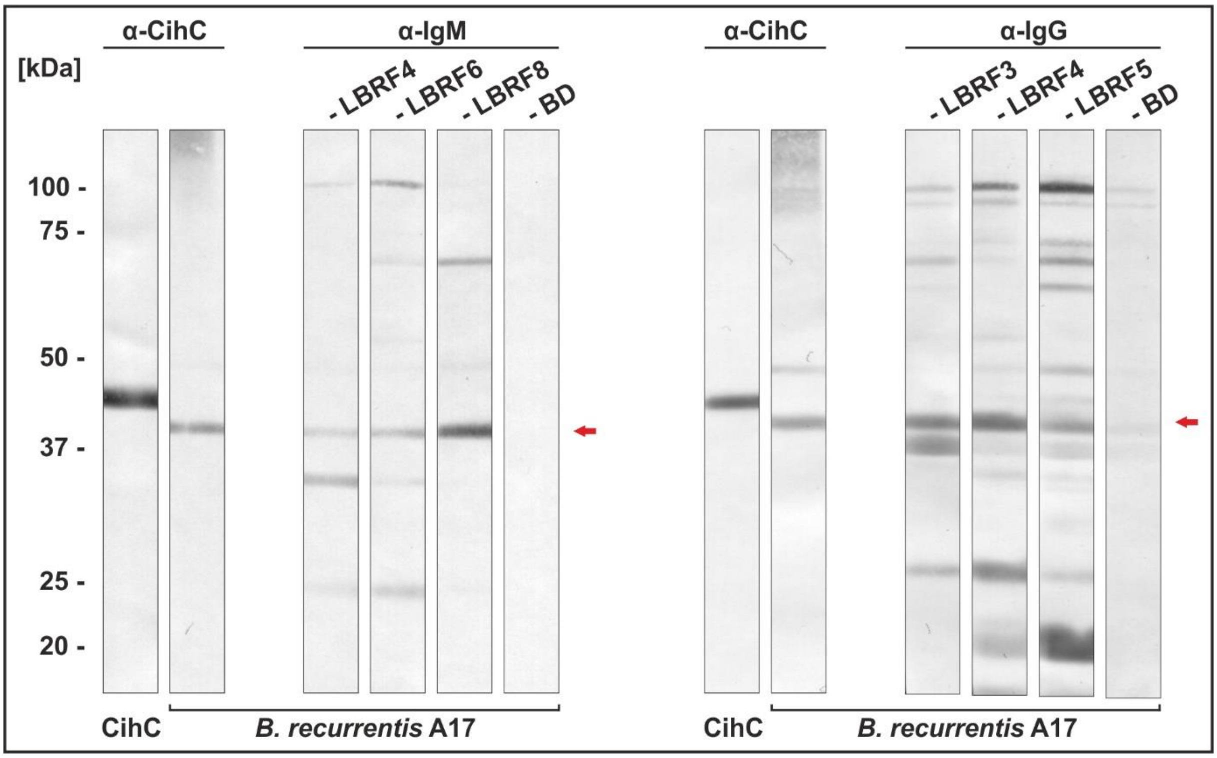

Supplementary figure 8. Determination of CihC in *B. recurrentis* A17 and detection of IgM and IgG responses to CihC. Purified His-tagged ChiC (500 ng) or whole cell lysate of *B. recurrentis* strain A17 (20 µg per lane) were separated by a Tris/Tricine SDS-PAGE and transferred to a nitrocellulose membrane. The membrane was cut into strips of which each was incubated with either a monoclonal anti-CihC antibody (clone BR2, Grosskinsky et al., 2010) or with different patient sera. IgM and IgG antibody responses were detected using LBRF sera LBRF4, LBRF6, and LBRF8, respectively as well as a BD serum sample (negative control). Signals corresponding to native ChiC were indicated by arrows. The mobilities of molecular mass standards in kDa are indicated on the left.
